# Zerumbone, a Southeast Asian Ginger Sesquiterpene, Induced Apoptosis of Pancreatic Carcinoma Cells through p53 Signaling Pathway

**DOI:** 10.1155/2012/936030

**Published:** 2012-01-29

**Authors:** Songyan Zhang, Qiaojing Liu, Yanju Liu, Hong Qiao, Yu Liu

**Affiliations:** ^1^Department of Hepatopancreatobiliary Surgery, The Third Affiliated Hospital of Harbin Medical University, Harbin 150040, China; ^2^Department of Laboratory Medicine, The Fourth Affiliated Hospital of Harbin Medical University, Harbin 150001, China; ^3^Department of Endocrinology, The Second Affiliated Hospital of Harbin Medical University, Harbin 150086, China

## Abstract

Pancreatic carcinoma is one common cancer with gradually increasing incidence during the past several decades. However, currently the candidate drugs to suppress pancreatic cancer remain lacking. This research was carried out to investigate if zerumbone, a natural cyclic sesquiterpene isolated from Zingiber zerumbet Smith, will produce the anticancer effects on pancreatic carcinoma cell lines. The results showed that zerumbone concentration, and time, dependently produced inhibitory actions on cell viability of PANC-1 cells. In addition, Hoechst 33342, AO/EB, TUNEL staining, and caspase-3 activity assay further showed that zerumbone induced apoptosis of PANC-1 cells. The expression of p53 protein was markedly upregulated, and the p21 level was also obviously elevated in zerumbone-treated PANC-1 cells. Moreover, ROS production was increased by about 149% in PANC-1 cells treated by zerumbone 30 *μ*M. Zerumbone also produced the same antitumor activity in pancreatic carcinoma cell lines SW1990 and AsPC-1. In summary, we found that zerumbone was able to induce apoptosis of pancreatic carcinoma cell lines, indicating to be a promising treatment for pancreatic cancer.

## 1. Introduction

As a crucial part of the digestive system, the incidence of pancreatic cancer is gradually increasing during the past decades all over the world. It was reported that approximately 37,000 individuals were diagnosed with pancreatic cancer in the United States [1]. The 5-year survival rate of patients with pancreatic cancer is less than 10%, and more than 30,000 people die from this cancer every year [2]. Pancreatic cancer remains one of the four or five most common causes of cancer mortality in developed countries. Currently, the therapeutic drugs for pancreatic cancers are lacking, and were hampered by their toxic actions on normal organs. Particularly, pancreatic cancer is seldom diagnosed during its early stages in clinics [2, 3]. Accordingly, developing the new drug and strategy to prevent or treat pancreatic cancer is an important mission.

**Figure 1:**
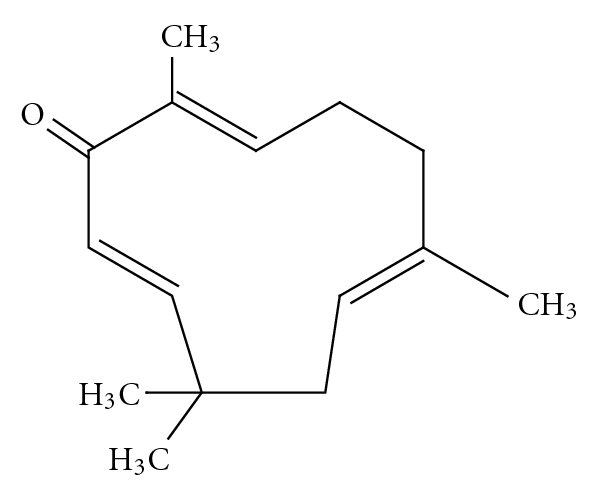
The chemical structure of zerumbone, a Southeast Asian ginger sesquiterpene.

Zingiber zerumbet Smith is one kind of plant growing mainly in Southeast Asia, which has been demonstrated to possess antinociceptive, anti-inflammatory, antiulcer, antihyperglycemic, and antiplatelet activities [4–7]. As a major compound extract, zerumbone is currently explored for its potential broad use on cancers, leukemia, as well as virus infection (Figure 1) [8–10]. Recently, several studies have shown that zerumbone also produced a variety of pharmacological effects, including antioxidants, antivirus, anti-inflammatory, hepatoprotection, antiplatelet aggregation, and antibacterial [8–13]. Recently, the increasing attention was paid to the anticancer actions of zerumbone. It was reported that zerumbone exhibited a strong ability to treat liver cancer, lung carcinogenesis, and leukemia through increasing the apoptosis and inhibiting the invasion [8, 12–15]. But, whether zerumbone played the inhibitory roles in pancreatic cancer cells remains unknown. The present study was undergone aiming to determine the antitumor role of zerumbone in pancreatic cancers.

## 2. Materials and Methods

### 2.1. Cell Culture

Human pancreatic carcinoma cell lines PANC-1 and SW1990 were cultured in Dulbecco's modified Eagle's medium (DMEM) containing penicillin (100 units/mL), streptomycin (100 *μ*g/mL), and L-glutamine (300 *μ*g/mL) supplemented with 10% fetal bovine serum (FBS). AsPC-1 cells were cultured in RPMI-1640 medium supplemented with 10% FBS. The culture condition for these cell lines was at 37°C in a humidified atmosphere of 5% CO_2_ and 95% air in a plastic flask. All cultured medium was changed twice every week.

### 2.2. Reagents

Zerumbone, DMSO, MTT (3-(4,5-Dimethylthiazol-2-yl)-2,5-Diphenyl Tetrazolium Bromide) kit, and other reagents, if not otherwise specified, were purchased from Sigma-Aldrich, St. Louis, MO, USA. Hoechst 33342 dye and Trizol were obtained from Invitrogen, Carlsbad, CA. In Situ Cell Death Detection Kit was bought from Roche, Penzberg, Germany (Catalog no. 11684795910). Caspase-3 activity colorimetric kit was purchased from R&D Systems Inc. (Biovision, Mountain View, USA). PA Lysis Buffer was obtained from Beyotime, Shanghai. The p53, p21 monoclonal, and PUMA polyclonal antibody were bought from Santa Cruz Biotechnology (Santa Cruz, CA). RNeasy Mini Kit and RNase-free DNase Set were obtained from Qiagen, Valencia, CA. TaqMan Reverse Transcription Reagents were purchased from Applied Biosystems, Foster City, CA. For all experiments of this study, DMSO was used to dissolve zerumbone. In order to avoid possible effects to these cells by DMSO, the volume of DMSO should not exceed 0.1% of the total volume (v/v).

### 2.3. Cell Proliferation Assay

The cellular viability of pancreatic cancer cells was determined by MTT (3-(4,5-Dimethylthiazol-2-yl)-2,5-Diphenyl Tetrazolium Bromide) assay. Briefly, the cells were collected and seeded in 96-well plates to attach overnight in Dulbecco's modified Eagle's medium (DMEM) supplemented with 10% fetal bovine serum (FBS). Human pancreatic cancer cells were rendered quiescent by incubation in serum-free media for 24 h. Then pancreatic cancer cells were incubated with zerumbone 3, 10, 30, and 100 *μ*M for 24 h or were cultured for 24, 48, and 72 h in the presence of 30 *μ*M, respectively. Then, the culture media were washed out and the fresh media containing 5 mg/mL MTT were added. The cells were continuously incubated at 37°C for an additional four hours. After this time, the media were washed out, and reduced MTT product (blue formazan product) was solubilized by adding 100 *μ*M DMSO to each wells. After agitation of these plates for 15 min, the optical density of the solubilized formazan product in each well was measured using a microplate reader at 570 nm with background subtraction at 650 nm. The experiment to observe different concentration of zerumbone on cellular viability of pancreatic cancer cells was carried out six times, and the experiment to study different incubation times of zerumbone on cellular viability was performed five times.

### 2.4. Acridine Orange/Ethidium Bromide (AO/EB) Staining

Morphological signs of apoptosis were detected by using acridine orange-ethidium bromide (AO/EB) staining in pancreatic cancer cells. The cells were incubated with zerumbone for 24 h. The procedure to perform AO/EB staining is just as described below. In order to staining the apoptotic cells, 10 *μ*L prepared AO/EB working solution (100 *μ*g/mL AO and 100 *μ*g/mL EB in PBS) was added to each well for 5 min. Then the pancreatic cancer cells were harvested and the apoptotic cells were counted under an inverted fluorescence microscope (Eclipse TE300, Nikon, Japan).

### 2.5. Hoechst 33342 Dye Staining

Morphological changes of apoptotic pancreatic cancer cells were evaluated by Hoechst 33342 staining. In brief, the cultured cells were planted in 6-well plates and then exposed to zerumbone treatment for 24 h. After being washed with PBS, the cancer cells were fixed in 4% paraformaldehyde for 30 min at room temperature. After being washed again with PBS, the fixed cells were stained with 20 *μ*g/mL Hoechst 33342 for 15 min at room temperature. The cells were imaged with fluorescence microscope.

### 2.6. Terminal Deoxynucleotidyl Transferase-Mediated dUTP Nick End Labeling (TUNEL) Assay

TUNEL assay was used to identify the apoptosis of pancreatic cancer cells. The cells were seeded in dishes, grown overnight, and subjected to zerumbone 3, 10, 30, and 100 *μ*M for 24 h. The staining of apoptotic cells was carried out using an In Situ Cell Death Detection Kit. In brief, after being washed twice with PBS, human pancreatic cancer cells were then fixed with 4% paraformaldehyde in PBS (pH 7.4) for 1 h at room temperature. The fixed cancer cells were permeabilised by incubation with 0.1% Triton X-100 in 0.1% sodium citrate for 2 min on ice. The cells were rinsed again with PBS and incubated with TUNEL reaction mixture for 1 h at 37°C in the dark. TUNEL staining of apoptotic cells was viewed under a fluorescence microscopy (Olympus, Tokyo, Japan).

### 2.7. Measurement of Reactive Oxygen Species (ROS)

To quantify intracellular ROS level, we used 2,7-dichlorodihydrofluorescein diacetate (H_2_DCF-DA) probe. The procedure for ROS measurement is as previously described [16]. Briefly, the cells were seeded and then were incubated with different concentration of zerumbone for 24 h. In order to detect the production of ROS, the pancreatic cancer cells were collected, washed twice with PBS, and loaded with H_2_DCF-DA 10 *μ*M by incubation for 30 min at 37°C. Fluorescence was measured by flow cytometry. The experiment with ROS assay was repeated four times.

### 2.8. Caspase-3 Activity Assay

To evaluate the caspase-3 activity, the cancer cells lysates were prepared after their respective treatment with zerumbone. The caspase-3 activity was determined by colorimetric kit. Then, assays were performed by incubating 20 mg of cell lysates with 200 mM chromogenic substrate (DEVD-pNA) in 100 mL reaction buffer. The cell lysate was incubated at 37°C for 2 h. Thereafter, the absorbance at 450 nm was measured to represent the release of chromophore p-nitroanilide (pNA). The experiment with caspase-3 assay was repeated three times.

### 2.9. Western Blot Analysis

For immunolabeling, the lysates were prepared after the cancer cells were subjected to their respective treatment with zerumbone. One hundred micrograms of each lysate were resolved by sodium dodecyl sulfate-polyacrylamide gel electrophoresis (SDSPAGE). After electrophoresis, the proteins were transferred onto a nitrocellulose membrane. After blocking with 5% nonfat dried milk and 0.05% Tween 20 in Tris-buffered saline (10 mM Tris, pH 8.0, 135 mM NaCl), the membranes were incubated overnight with the relevant primary antibody followed by the incubation with horseradish peroxidase-conjugated immunoglobulin G (IgG). The blots were then visualized by using Odyssey v1.2 software. The experiments were repeated three times.

### 2.10. Quantitative Real-Time PCR Analysis

According to the guideline of the manufacturer, the total RNA from pancreatic cancer cells was isolated by Trizol and purified by RNeasy Mini Kit and RNase-free DNase Set. Total RNA from pancreatic cancer cells was subjected to first-strand cDNA synthesis using TaqMan Reverse Transcription Reagents. The method to determine miR-34 mRNA level in cancer cells is just as described previously [17]. Relative mRNA for miR-34 was calculated by the comparative CT method (DDCT) using U6 as an endogenous control and untreated samples as the calibrator.

### 2.11. Statistical Analysis

All data was presented as mean ± S.E.M. Statistical analysis was performed to determine the significance of differences among groups using ANOVA. All statistical analysis was performed using the SPSS 13.0 software for Windows. Statistical significance was initially set at *P* < 0.05.

## 3. Results

### 3.1. Zerumbone Reduced Cellular Viability of PANC-1 Cells

**Figure 2:**
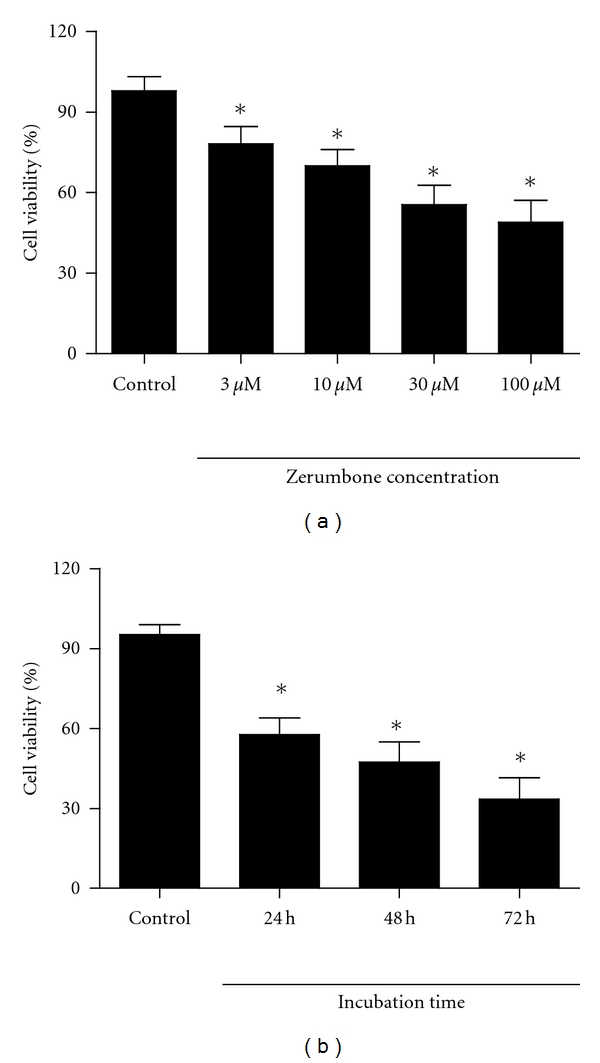
Effects of zerumbone on the cellular viability of PANC-1 cells. (a) The cellular viability of PANC-1 was significantly reduced by zerumbone 3, 10, 30, and 100 *μ*M after 24 h incubation. *n* = 6 independent experiments. (b) Zerumbone obviously decreased the cellular viability of PANC-1 cells in a time-dependent manner. *n* = 5 independent experiments, *P < 0.05 versus Control.

The effects of zerumbone on the proliferation of PANC-1 cells were measured by the MTT assay. As displayed in Figure 2(a), the exposure of PANC-1 cells to zerumbone 3 *μ*M, 10 *μ*M, 30 *μ*M, and 100 *μ*M for 24 h resulted in a significant reduction of cellular viability, compared with untreated cells (*P* < 0.05). Zerumbone 3 *μ*M, 10 *μ*M, 30 *μ*M, and 100 *μ*M decreased the viability of PANC-1 cells from 97.9 ± 5.3 to 78.2 ± 6.4, 70.1 ± 6.0, 55.6 ± 7.2, and 49.1 ± 8.1, respectively (*P* < 0.05).  Figure 2(b) showed that cellular viability of PANC-1 cells after exposure to zerumbone 30 *μ*M for 24 h, 48 h, and 72 h was decreased from 95.3 ± 3.8 to 57.8 ± 6.2, 47.4 ± 7.5, and 33.6 ± 7.9. The results suggest that zerumbone reduces the proliferation of PANC-1 in a concentration- and time-dependent manner (*P* < 0.05).

### 3.2. Apoptosis of PANC-1 Cells Was Induced by Exposure to Zerumbone

**Figure 3:**
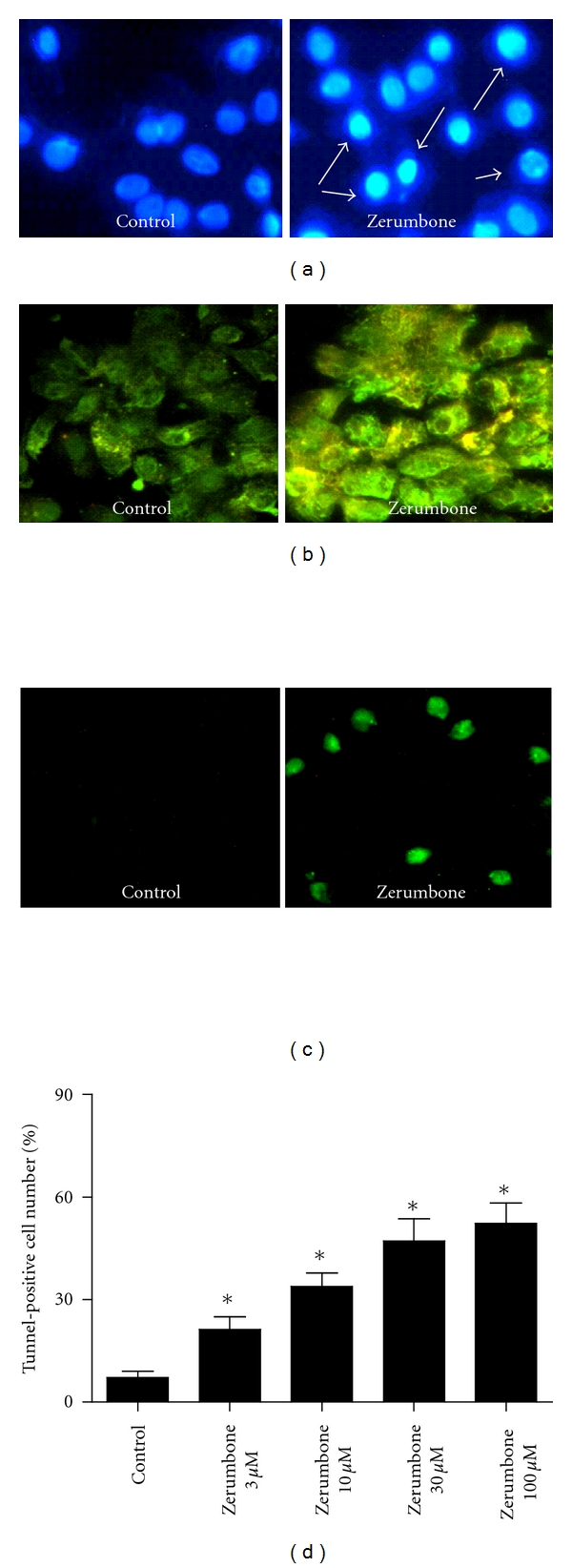
Effects of zerumbone on apoptosis of PANC-1 cells. (a) Hoechst 33342 staining of the apoptosis of PANC-1 in the presence of zerumbone 30 *μ*M. (b) Effects of zerumbone 30 *μ*M on apoptosis of PANC-1 cells were identified by AO/EB staining. (c) and (d) TUNEL-positive cells were viewed in the presence of zerumbone 30 *μ*M. *n* = 3 independent experiments, *P < 0.05 versus Control.

We further used Hoechst 33342 and AO/EB staining to determine the effects of zerumbone on the apoptosis of PANC-1 cells. As demonstrated in Figures 3(a) and 3(b), zerumbone-treated PANC-1 cells exhibited obvious apoptotic morphological changes in the nuclear chromatin, such as cell shrinkage, chromatin condensation, and cell nuclear fragmentation. By contrast, PANC-1 cells without zerumbone treatment presented the intact nuclear architecture (Figure 3(b)). As shown in Figure 3(c), TUNEL-positive staining could be detected more significantly in PANC-1 cells pretreated by zerumbone than in untreated PANC-1 cells. Zerumbone 3 *μ*M, 10 *μ*M, 30 *μ*M, and 100 *μ*M significantly increased the number of TUNEL-positive PANC-1 cells from 7.1 ± 1.9% to 21.3 ± 3.6, 33.8 ± 4.0, 47.1 ± 6.6 and 52.3 ± 5.9 after 24 h incubation (*P* < 0.05).

### 3.3. Zerumbone Increased the Activity of Caspase-3 and ROS in PNAC-1 Cells

**Figure 4:**
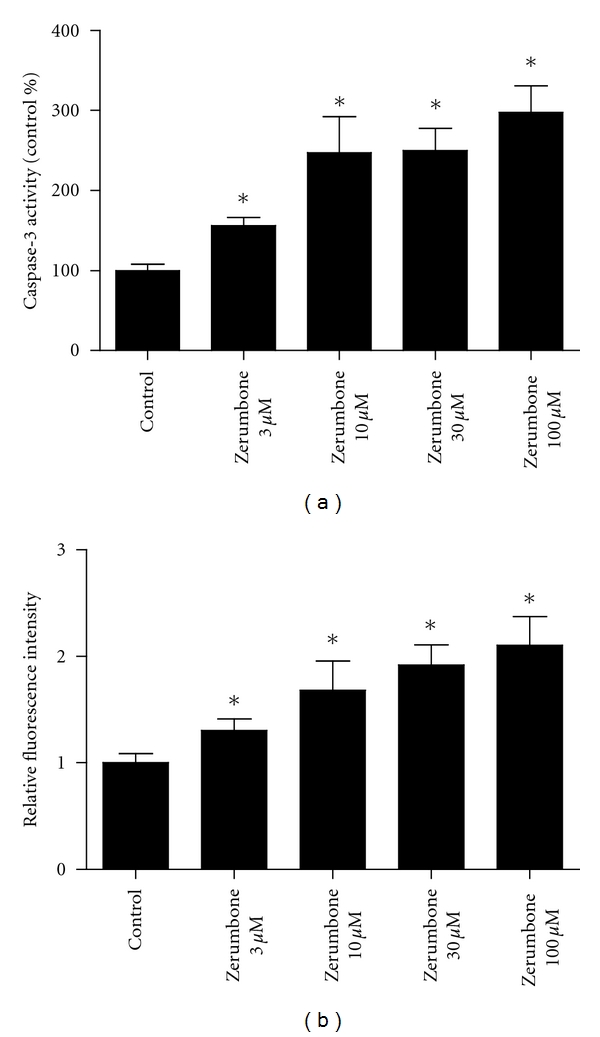
Effects of zerumbone on the caspase-3 activity and the generation of ROS production of PNAC-1 cells. (a) Zerumbone increased the caspase-3 activity of PANC-1 cells. *n* = 3 independent experiments. (b) Zerumbone 30 *μ*M increased the fluorescence density of ROS. Zerumbone increased the ROS generation in a concentration-dependent manner. *n* = 4 independent experiments, *P < 0.05 versus Control.

The effect of zerumbone on the activity of caspase-3 in PANC-1 cells was further investigated. As illuminated in Figure 4(a), the exposure of PANC-1 cells to zerumbone 3 *μ*M, 10 *μ*M, 30 *μ*M, and 100 *μ*M markedly increased the activity of caspase-3 by approximately 56%, 147%, 149%, and 197%, respectively (*P* < 0.05). These results further confirmed that zerumbone induced apoptosis of PANC-1 cells. Then, we explored the influences of zerumbone on the production of ROS. PANC-1 cells were exposed to zerumbone 3 *μ*M, 10 *μ*M, 30 *μ*M, and 100 *μ*M for 24 h and analyzed for the production of ROS by fluorescence microscopy.  Figure 4(b) demonstrated the fluorescence image of ROS in the absence and presence of zerumbone in PANC-1 cells. The generation of ROS was increased by zerumbone in a concentration-dependent manner (*P* < 0.05).

### 3.4. Effects of Zerumbone on the Expression of p53 and miR-34

**Figure 5:**
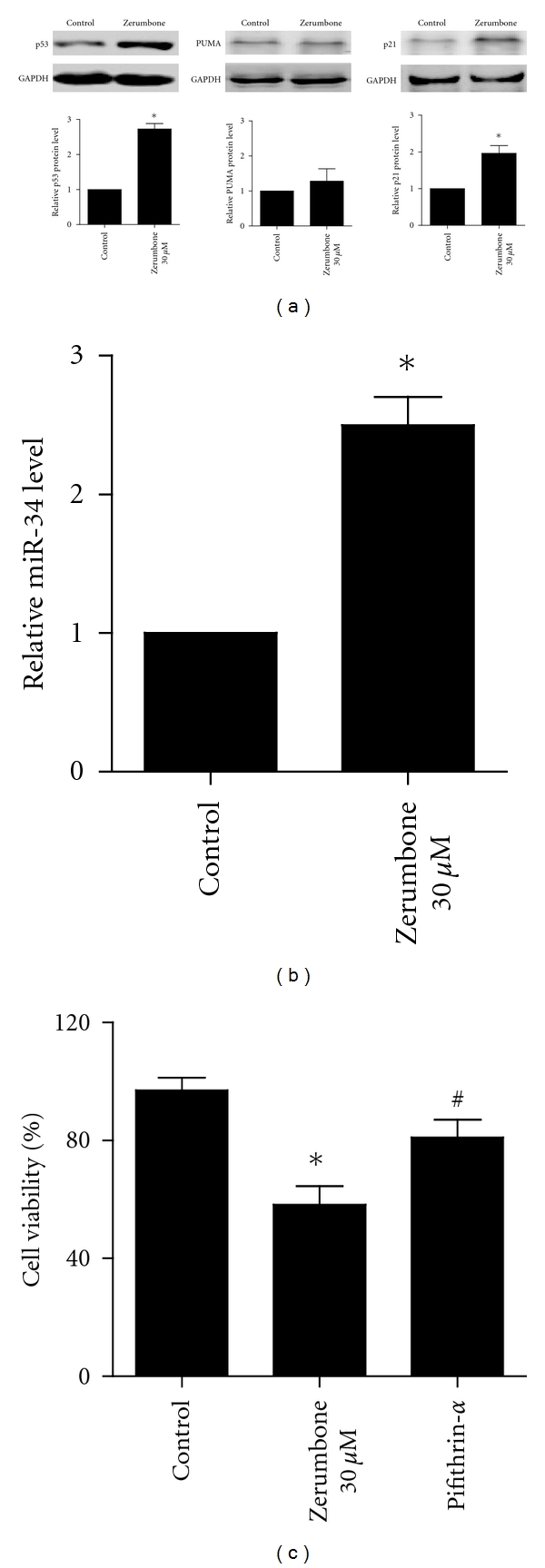
Effects of zerumbone on the expression of p53 and miR-34. (a) Zerumbone 30 *μ*M markedly inhibited the p53, p21, and PUMA protein expression. (b) Zerumbone 30 *μ*M decreased the miR-34 level. (c) The p53 specific inhibitor pifithrin *α* reversed the inhibitory influences of zerumbone on cellular viability. *n* = 3 independent experiments, *P < 0.05 versus Control, ^#^ *P* < 0.05 versus Zerumbone.

We further investigate whether zerumbone plays a regulatory role in the expression of p53 and miR-34. As displayed in Figure 5(a), pretreatment with zerumbone 30 *μ*M significantly increased the expression of p53 protein in PANC-1 cells (*P* < 0.05). In agreement, miR-34 level was also augmented in zerumbone-treated PANC-1 cells (*P* < 0.05) (Figure 5(b)). These results imply that p53 signal pathway is involved in the apoptosis of PANC-1 cells induced by zerumbone. Moreover, the effects of zerumbone on p21 and PUMA protein were investigated and the results showed that PUMA was not affected but p21 was significantly upregulated, indicating that p53 and p21 signal pathway was activated after treatment with zerumbone (Figure 5(a)) (*P* < 0.05). We further investigate the effects of the p53-specific inhibitor pifithrin-*α* on zerumbone-induced decrease of cellular viability in PANC-1 (Figure 5(c)). The results showed that pifithrin-*α* 20 *μ*M reversed the inhibitory role of zerumbone 30 *μ*M in cellular viability of PANC-1, indicating that zerumbone exerts antitumor effects through p53-dependent manner.

### 3.5. Zerumbone Induced Apoptosis in SW1990 and AsPC-1 Cells

**Figure 6:**
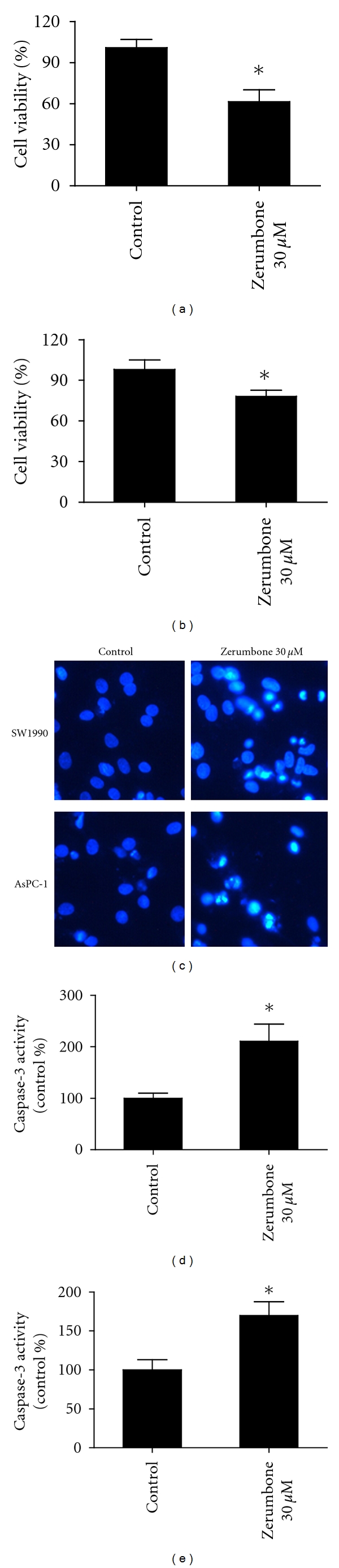
Zerumbone induced apoptosis in SW1990 and AsPC-1 cells. (a) Zerumbone 30 *μ*M significantly decreased the cellular viability of SW1990 after 24 h incubation. (b) The cell viability of AsPC-1 cells was also strongly inhibited in the present of Zerumbone 30 *μ*M. (c) Hoechst 33342 staining of SW1990 and AsPC-1 cells in the absence and presence of zerumbone 30 *μ*M. (d) Zerumbone 30 *μ*M increased the caspase-3 activity in SW1990 cells. (e) Zerumbone 30 *μ*M also enhanced the caspase-3 activity in AsPC-1 cells. *n* = 3 independent experiments, *P < 0.05 versus Control.

We also studied the antitumor effects of zerumbone on another two pancreatic cancer cell lines SW1990 and AsPC-1. Figures 6(a) and 6(b) showed that zerumbone 30 *μ*M markedly inhibited cellular viability of SW1990 and AsPC-1 after 24 h incubation (*P* < 0.05). Furthermore, Hoechst 33342 staining displayed that the exposure to zerumbone 30 *μ*M for 24 h induced obvious apoptotic morphological changes in the nuclear chromatin in SW1990 and AsPC-1 (Figure 6(c)). Figures 6(d) and 6(e) showed that zerumbone 30 *μ*M increased the caspase-3 activity in both SW1990 and AsPC-1. These findings suggest the antitumor role of zerumbone in SW1990 and AsPC-1 cell lines.

## 4. Discussion

It was demonstrated in this study that exposure to zerumbone resulted in apoptosis of PANC-1 cells through p53 signal pathway. The present research offers us a new understanding about the molecular mechanisms of antitumor actions of zerumbone on pancreatic cancer.

A large body of evidence demonstrated that apoptosis is a normal component of the development and health of multicellular organisms and also is a key way to clear the unnecessary cells [18, 19]. Notably, apoptosis is more important in understanding cancer, because cancer cells have developed a way to avoid apoptosis [20]. Thus, cancer is often characterized by too little apoptosis and too much proliferation of cells. To promote apoptosis and inhibit proliferation of cancer cells has been suggested as a therapeutic approach.

Zerumbone is a sesquiterpene phytochemical from a type of edible ginger known as “Zingiber zerumbet Smith” grown in Southeast Asia or “Zingiber aromaticum” [4–9]. In several studies, zerumbone has been showed to play an antitumor role in liver cancer, leukemia, and lung carcinogenesis, which was considered as a promising therapeutic drug for cancers [8–15]. For example, zerumbone was reported to induce G2/M cell cycle arrest and apoptosis in leukemia cells through a Fas- and mitochondria-mediated pathway [12]. In addition, zerumbone also could effectively suppress mouse colon and lung carcinogenesis through multiple modulatory mechanisms of growth, apoptosis, inflammation, and expression of NFkappaB and HO-1 after dietary administration [13]. Zerumbone was shown to strongly inhibit the proliferation of liver cancer cells and enhance the apoptosis [15]. However, the information about the therapeutic effects of zerumbone on pancreatic cancer cells is unavailable. In this study, we uncover for the first time that zerumbone-treated pancreatic cancer cells exhibited a decreased proliferation and increased apoptosis, which is characterized by the formation of apoptotic bodies, condensed nuclei, and the increased activity of caspase-3. The present study therefore offered a new possible application of zerumbone in the treatment of pancreatic cancer.

It is well documented that p53 plays an important role in the control of cell cycle and apoptosis [20]. As a tumor suppressor, p53 plays a more crucial role in preventing tumor development [21]. It is considered responsible for a range of potentially oncogenic stresses by activating antitumor mechanisms, most notably cell cycle arrest and apoptosis. The present study displayed that p53 was significantly increased in zerumbone-treated PANC-1 cells. It suggests that p53 may contribute to the inhibition of the apoptosis of pancreatic cancer cells by zerumbone.

A new component of p53 signaling pathway was recently uncovered, and it was showed that the activation of endogenous p53 induced the upregulation of miR-34 expression and p21, suggesting that miR-34 is a direct target of p53 [22]. Furthermore, it was previously reported that the overexpression of miR-34a led to the growth arrest and apoptosis in neuroblastoma cells by silencing the expression of E2f3 [23]. We found that miR-34 and p21 were obviously increased in zerumbone-treated PANC-1 cells, indicating that p53 signal pathway is activated by zerumbone.

Reactive oxygen species (ROS) are a variety of molecules and free radicals derived from molecular oxygen, which was constantly generated and eliminated in the biological system, and have important roles in cell signaling and homeostasis [24]. Excessive amounts of ROS can cause oxidative damage to lipids, proteins, and DNA leading to tumorigenesis or cell death. Although the use of antioxidants in humans for cancer prevention remains controversial, increasing evidence supported that the increase of ROS generation contributed to the treatment of cancer cells. Reactive oxygen species are suggested as downstream mediators of p53-dependent apoptosis [25, 26]. The cells sensitive to p53-mediated apoptosis promoted the generation of ROS, whereas cells resistant to p53 failed to produce ROS [25]. We found that zerumbone exerted a facilitated role in the production of ROS in a concentration-dependent manner, which is at least in part responsible for its pharmacological actions on PANC-1 cell lines.

In summary, it was uncovered in our study that zerumbone induced apoptosis in pancreatic carcinoma cells through p53 signal pathway. This finding indicates zerumbone, a sesquiterpene in subtropical ginger, as a new therapeutic candidate for pancreatic cancer.
